# Apigenin and Luteolin Attenuate the Breaching of MDA-MB231 Breast Cancer Spheroids Through the Lymph Endothelial Barrier *in Vitro*

**DOI:** 10.3389/fphar.2018.00220

**Published:** 2018-03-14

**Authors:** Junli Hong, Adryan Fristiohady, Chi H. Nguyen, Daniela Milovanovic, Nicole Huttary, Sigurd Krieger, Junqiang Hong, Silvana Geleff, Peter Birner, Walter Jäger, Ali Özmen, Liselotte Krenn, Georg Krupitza

**Affiliations:** ^1^Department of Pharmacognosy, Faculty of Life Sciences, University of Vienna, Vienna, Austria; ^2^School of Pharmacy, Nanjing Medical University, Nanjing, China; ^3^Clinical Institute of Pathology, Medical University of Vienna, Vienna, Austria; ^4^Department for Clinical Pharmacy and Diagnostics, Faculty of Life Sciences, University of Vienna, Vienna, Austria; ^5^Faculty of Pharmacy, Halu Oleo University, Kendari, Indonesia; ^6^Department of Medical Oncology, The 188th Hospital of People’s Liberation Army of China, Chaozhou, China; ^7^Department of Biology, Faculty of Science and Art, Adnan Menderes University, Aydin, Turkey

**Keywords:** intravasation, 3D model, flavonoids, FAK, MMP1, CYP1A1, Ca^2+^ release

## Abstract

Flavonoids, present in fruits, vegetables and traditional medicinal plants, show anticancer effects in experimental systems and are reportedly non-toxic. This is a favorable property for long term strategies for the attenuation of lymph node metastasis, which may effectively improve the prognostic states in breast cancer. Hence, we studied two flavonoids, apigenin and luteolin exhibiting strong bio-activity in various test systems in cancer research and are readily available on the market. This study has further advanced the mechanistic understanding of breast cancer intravasation through the lymphatic barrier. Apigenin and luteolin were tested in a three-dimensional (3-D) assay consisting of MDA-MB231 breast cancer spheroids and immortalized lymph endothelial cell (LEC) monolayers. The 3-D model faithfully resembles the intravasation of breast cancer emboli through the lymphatic vasculature. Western blot analysis, intracellular Ca^2+^ determination, EROD assay and siRNA transfection revealed insights into mechanisms of intravasation as well as the anti-intravasative outcome of flavonoid action. Both flavonoids suppressed pro-intravasative trigger factors in MDA-MB231 breast cancer cells, specifically MMP1 expression and CYP1A1 activity. A pro-intravasative contribution of FAK expression in LECs was established as FAK supported the retraction of the LEC monolayer upon contact with cancer cells thereby enabling them to cross the endothelial barrier. As mechanistic basis, MMP1 caused the phosphorylation (activation) of FAK at Tyr397 in LECs. Apigenin and luteolin prevented MMP1-induced FAK activation, but not constitutive FAK phosphorylation. Luteolin, unlike apigenin, inhibited MMP1-induced Ca^2+^ release. Free intracellular Ca^2+^ is a central signal amplifier triggering LEC retraction through activation of the mobility protein MLC2, thereby enhancing intravasation. FAK activity and Ca^2+^ levels did not correlate. This implicates that the pro-intravasative contribution of FAK and of Ca^2+^ release in LECs was independent of each other and explains the better anti-intravasative effects of luteolin *in vitro*. In specific formulations, flavonoid concentrations causing significant anti-intravasative effects, can certainly be achieved *in vivo*. As the therapeutic strategy has to be based on permanent flavonoid treatment both the beneficial and adverse effects have to be investigated in future studies.

## Introduction

Species of the *Scrophularia* family are used in ethno-medicine since ancient times (Galindez et al., 2002). In the search for anti-metastatic natural compounds, extracts of six *Scrophularia* species were tested. For a methanolic extract from *S. lucida* a significant anti-intravasative activity was discovered (Giessrigl et al., 2012). The chloroform fraction of the methanolic extract inhibited *in vitro* intravasation of breast cancer spheroids most effectively. The major compound of the most active sub-fraction was hispidulin which, as pure compound, attenuated this early stage of the metastatic process significantly (Lewenhofer et al., 2018, same issue). Therefore, we decided to test two structurally related flavonoids, luteolin and apigenin, in in-depth mechanistic studies. These flavonoids are present in many medicinal plants used in the traditional medicine of Central and Eastern Europe. One of the oldest references to a healing plant in occidental literature can be found in Homer’s *Iliad* (book XI, line 822 et seq.; Murray, 1924). There is said that ’Aχιλλεύς used a bitter, astringent root to treat wounds. This background in mythology led to the name of a very important medicinal plant: *Achillea*. In fact, this plant is used to treat, among other ailments, bleeding disorders and bruises (Akram, 2013). Later, *Achillea* sp. (“Achilleios”) was also listed in *De Materia Medica*; Dioscurides, 1st century A.D. and used by Classical Greeks, in the Roman Empire, the Byzantium, and the Middle Ages up until our present day. *A. millefolium* is very rich in glycosides of luteolin and apigenin, which are easily metabolized into the respective genins (Benedek et al., 2007; Benetis et al., 2008). Many other apigenin- and luteolin-rich plant genera are also listed in *De Materia Medica*: *Cynara, Apium, Allium, Petroselinum, Punica, Thymus, Solidago, Chamaemelum, Viola*, and *Vitis* (the entire 5^th^ book is dedicated to *Vitis*).

Triple negative breast cancer (TNBC) comprises about 15% of all breast cancer types. In general, TNBC responds well to chemotherapy as long it has not disseminated to other organs. However, as soon as TNBC metastasizes the success of drug treatment is very limited. This implicates that TNBC, in addition to conventional therapy, needs a metastasis-preventive approach to maintain a tumor state associated with a better prognosis. At an early stage of metastasis breast cancer cells intravasate the lymphatic vasculature and colonize lymph nodes. Importantly, the “lymph node status” is a prognostic breast tumor marker (Sobin et al., 2009). Hence, we hypothesized that compounds which prevent intravasation may interfere with tumor progression such as lymph node- and distant organ metastasis. As such a preventive concept for TNCB has to be based on long term drug administration, the therapeutic regimen has to be mild and rather free of side effects to allow continuous treatment. Luteolin inhibited proliferation and induced apoptosis of the highly metastatic TNBC cell line MDA-MB231 (Attoub et al., 2011). Anti-invasive/anti-metastatic activities of luteolin (Tsai et al., 2016; Lin et al., 2017) and the structurally related analog apigenin (Lindenmeyer et al., 2001; Qin et al., 2016; Zhao et al., 2017) have also been demonstrated in various cancer cell lines. As these flavonoids are also wide-spread constituents in fruits and vegetables they are considered free of adverse effects. This tempted us to investigate luteolin and apigenin in terms of their anti-intravasative properties. In the daily diet, the concentration of flavonoids is too low to show tumor preventive effects (Vogiatzoglou et al., 2015). However, apigenin and luteolin are also commercially available as food supplements with rather high concentrations (40–200 mg apigenin/capsule; 50 mg luteolin/capsule) and may provide a preventive option.

The TNCB cell line MDA-MB231 over-expresses the pro-metastatic matrix metalloprotease 1 (MMP1), and inhibition of MMP1 attenuates tumor intravasation (Nguyen et al., 2015). Furthermore, MDA-MB231 cells secrete the pro-intravasative arachidonic acid metabolite 12(S)-HETE (Nguyen et al., 2016a), which is synthesized by CYP1A1. Therefore, we investigated whether luteolin and apigenin interfered with MMP1 expression and CYP1A1 activity in MDA-MB231 cells.

MMP1 and 12(S)-HETE activate the PAR1 and the 12(S)-HETE receptor, respectively, in LECs, which both trigger the release of Ca^2+^, causing the rapid retraction of LECs. This opens gaps in the monolayer [circular chemorepellent-induced defects (CCIDs)] through which tumor cells cross the endothelial barrier (Nguyen et al., 2015, 2016b). Hence, the influence of apigenin and luteolin on levels of intracellular Ca^2+^ in LECs was investigated. The expression of FAK in LECs seems to be crucial for rapid cell movement (Desai et al., 2009) as observed during CCID formation. Thus, FAK expression and activity were studied as well.

## Materials and Methods

### Antibodies and Reagents

Polyclonal rabbit anti-focal adhesion kinase (FAK) and polyclonal rabbit anti-phospho-Tyr397-FAK were purchased from Cell Signaling (Danvers, MA, United States), polyclonal rabbit anti-MMP1 from Abcam (Cambridge, United Kingdom) and monoclonal anti-β-actin from Sigma (Munich, Germany). Polyclonal rabbit anti-mouse, anti-goat and anti-rabbit IgGs were acquired from Dako (Glostrup, Denmark). Apigenin and luteolin were purchased from Sigma (Munich, Germany) and human recombinant MMP1 from Sigma-Aldrich (SRP3117, St. Louis, MO, United States). U73122 was obtained from Calbiochem (Darmstadt, Germany), BAPTA-AM was from Santa Cruz Biotechnology (Heidelberg, Germany).

siRNAs targeting human MLC2 (MYL2; SMART pool, ON-TARGET PLUS, Cat. No.: L-011087000005, siRNAs targeting human FAK (SMART pool, ON-TARGET PLUS, Cat. No.: L-003164000005) were ordered from Dharmacon (Gene Expression and Gene Editing, GE Healthcare, Lafayette, CO, United States). Non-targeting (n.t.) control siRNA (Silencer^®^ Select Negative Control No. 1 siRNA, Cat. No.: 4390843) was purchased from Ambion (Life Technologies, Carlsbad, CA, United States). All siRNAs were re-suspended in RNAse-free water to yield a stock concentration of 20 μM.

### Cell Culture

Human MDA-MB231 breast cancer cells from the American Type Culture Collection (ATCC, Rockville, MD, United States) were grown in MEM medium supplemented with 10% fetal calf serum (FCS), 1% penicillin/streptomycin (PS) and 1% non-essential amino acids (Gibco, Invitrogen, Karlsruhe, Germany). Telomerase immortalized human lymph endothelial cells (LECs) were grown in EGM2 MV (Clonetics CC-4147, Allendale, NJ, United States). The cells were kept at 37°C in a humidified atmosphere containing 5% CO_2_. For CCID formation assays, LECs were stained with CellTracker^TM^ green purchased from Invitrogen (Karlsruhe, Germany).

### Spheroid Formation

MDA-MB231 cells (input of 6.000 cells per spheroid) were transferred to 30 mL serum free MEM medium containing 6 mL of a 1.6% methylcellulose solution (0.3% final concentration; Cat. No.: M-512, 4000 centipoises; Sigma-Aldrich, Munich, Germany). 150 μL of cell suspension were transferred to each well of a 96-well plate (Greiner Bio-one, Cellstar 650185, Kremsmünster, Austria) to allow spheroid formation within 48 h.

### CCID (Circular Chemorepellent Induced Defect) Assay

In this assay, the sizes of the cell free areas (CCIDs) formed in the endothelial monolayer underneath the tumor spheroids, were measured. MDA-MB231 spheroids were washed in PBS and transferred to CellTracker^TM^ (green)-stained LEC monolayers seeded into 24-well plates (Costar 3524, Sigma-Aldrich, Munich, Germany) in 1 mL EGM2 MV medium. After 4 h of incubation, the CCID areas in the LEC monolayers underneath the MDA-MB231 spheroids were photographed using an Axiovert (Zeiss, Jena, Germany) fluorescence microscope. CCID areas were calculated using the Zen Little 2012 (Zeiss, Jena, Germany). For each treatment and control the CCID size of at least 25 spheroids (unless otherwise specified) was measured.

### SDS Gel Electrophoresis and Western Blotting

MDA-MB231 cells and LECs were grown in T-25 tissue culture flasks (Nunc, Roskilde, Denmark) to 80% confluence and then pre-treated with the flavonoids for 0.5, 1, 2, and 4 h. Then, cells were processed for SDS gel electrophoresis and Western blotting as described before (Nguyen et al., 2015). Chemo-luminescence was developed by Amersham ECL prime Kit (GE Healthcare, Freiburg, Germany) and detected using a Lumi-Imager F1 Workstation (Roche, Basel, Switzerland). Densitometry of the Western blots was analyzed with the Image-J software (National Institutes of Health, Bethesda, MD, United States).

### Ethoxyresorufin-*O*-Deethylase (EROD) Assay Selective for CYP1A1/A2 Activity

MDA-MB231 cells were grown in phenol red-free DMEM/F12 medium (Gibco, Karlsruhe, Germany) containing 10% FCS and 1% PS (Invitrogen, Karlsruhe, Germany). Before treatment, the cells were transferred to DMEM/F12 medium supplemented with 10% charcoal-stripped FCS (PAN Biotech, Aldenbach, Germany) and 1% PS. After 4 h of treatment with apigenin and luteolin CYP1A1 activity was measured with minor modifications as previously described (Teichmann et al., 2014). Briefly, ethoxyresorufin (final concentration 5.0 μM, Sigma-Aldrich, Munich, Germany) was added, 0.4 mL aliquots of the medium were collected after 180 min and the formation of resorufin was analyzed by spectro-fluorometry (PerkinElmer LS50B, Waltham, MA, United States) at an excitation wavelength of 530 nm and an emission wavelength of 585 nm.

### Intracellular Ca^2+^ Assay

Free intracellular Ca^2+^ levels were measured using the FluoForte^TM^ Calcium Assay Kit (Enzo Life Sciences, Ann Arbor, MI, United States). 8 × 10^3^ LECs/well/100 μL EGM2 medium were seeded into 96-well black-wall clear-bottom plates (Nunc, Thermo Fisher Scientific, Rochester, NY, United States) and after 24 h LECs were pre-treated with apigenin, luteolin or DMSO for 1 h. Then, medium was removed and 100 μL FluoForte^TM^ Dye-loading (containing apigenin, luteolin, or DMSO) were added to each well. Cells were further incubated for 45 min at 37°C followed by 15 min at room temperature. Then, cells were stimulated with 1 μM 12(S)-HETE for 5 min, and fluorescence was measured with a fluorescence plate reader at 490/525 nm.

### Transfection of LEC Monolayer

Lymph endothelial cells were seeded in 24-well plates (1 mL/well) and grown in EGM2 medium. Transfections were performed at a confluence of 70–80%. 0.75 μg siRNA and 6 μL HiPerFect Transfection Reagent (Cat. No.: 301705; Qiagen, Hilden, Germany) were mixed in 100 μL serum-free medium and incubated for 30 min at room temperature to allow the formation of transfection complexes. Cell culture medium was discarded and 500 μL of fresh EGM2 medium were added into each well. Then, the transfection complexes were added drop-wise to the cells (to a final siRNA concentration of 100 nM) and incubated for 24 h at 37°C. After 24 h, the medium was replaced and cells were incubated for another 24 h to recover. The LEC monolayers were used for CCID assays or isolated RNA for qPCR.

### Statistical Analysis

For statistical analyses Excel 2013 software and Prism 6 software packages (GraphPad, San Diego, CA, United States) were used. The values were expressed as mean ± SEM, and the Student’s *t*-test and ANOVA with Tukey’s post-test was used to compare differences between control samples and treatment groups as well as differences among treatment groups. The statistical significance level was set to *p* < 0.05.

## Results

### Apigenin/Luteolin Inhibit CCID Formation Induced by MDA-MB231 Spheroids

**FIGURE 1:**
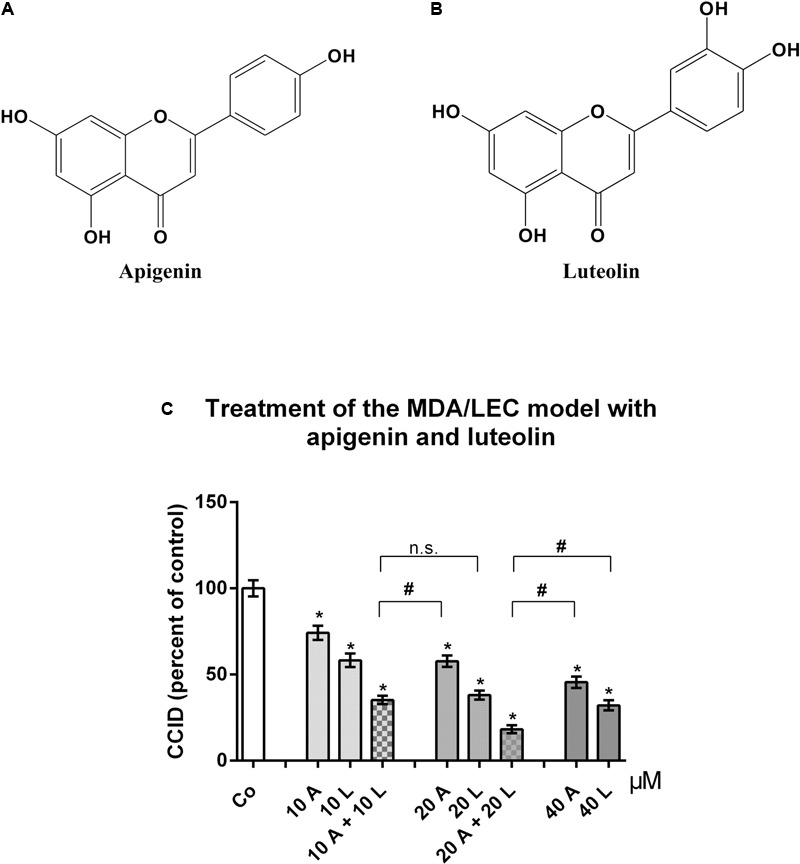
Effects of flavonoid combinations on CCID formation. Chemical structures of **(A)** apigenin and **(B)** luteolin. **(C)** MDA-MB231 spheroids were placed on LEC monolayers and treated with solvent (DMSO, Co), or with 10, 20, and 40 μM apigenin (A) or luteolin (L), or combinations of 10 and 20 μM A and L (solvent was kept constant) for 4 h, after which CCID areas were measured using an Axiovert microscope and Zen little 2012 software. Three independent experiments with at least five replicates were analyzed. Error bars indicate means ± SEM and asterisks and hash significances (*p* < 0.05; *t*-test).

Breast cancer cells have to intravasate the lymphatic barrier to colonize lymph nodes, and the CCID *in vitro* assay realistically resembles the intravasation of tumor spheroids through the lymphatic vasculature (Kerjaschki et al., 2011). Treatment of the three-dimensional MDA-MB231/LEC model with apigenin (**Figure 1A**) and luteolin (**Figure 1B**) dose dependently inhibited CCID formation in the LEC monolayer, with luteolin showing a stronger effect than apigenin. Simultaneous treatment with 20 μM luteolin and 20 μM apigenin synergized and inhibited CCID formation significantly stronger than 40 μM luteolin or 40 μM apigenin, respectively, thus suggesting that luteolin and apigenin interfered with distinct pro-intravasative mechanisms (**Figure 1C**).

### Apigenin/Luteolin Inhibit MMP1 Expression and CYP1A1 Activity in MDA-MB231 Cells

**FIGURE 2:**
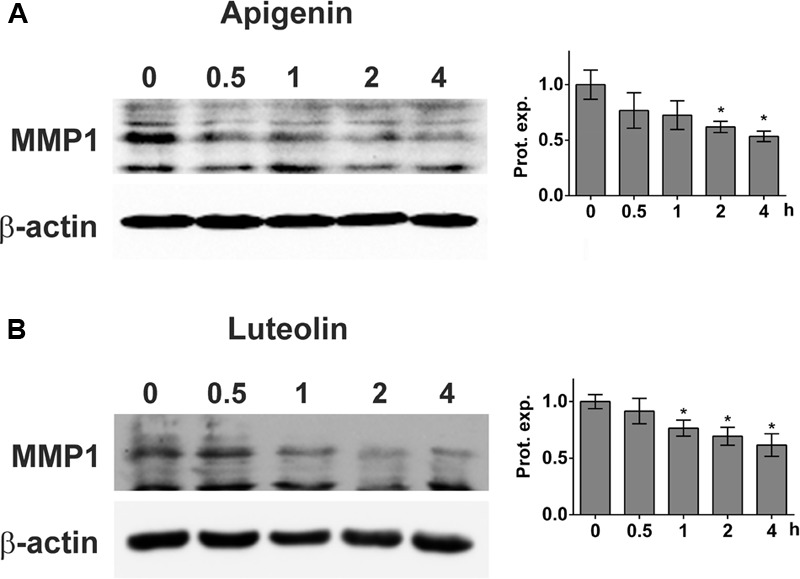
Inhibition of MMP1 expression upon flavonoid treatment. MDA-MB231 cells were grown to ∼80% confluence and then treated with solvent (0), or **(A)** 20 μM apigenin, or **(B)** 20 μM luteolin for 0.5, 1, 2, and 4 h. Then, cells were lysed, proteins separated by SDS gel electrophoresis and subjected to Western blotting using the indicated antibodies. Staining with Ponceau S and immunoblotting with anti-β-actin antibody controlled equal sample loading. Relative protein expression levels (Prot. expr.) are shown to the right of the blots. Densitometries are means ± SEM from at least three experiments (asterisks indicate significances, *p* < 0.05; *t*-test) and the Western blot images are representatives for illustration.

MDA-MB231 cell spheroids express MMP1, which is one mechanism causing CCIDs in the adjacent LEC monolayer (Nguyen et al., 2015). Thus, the expression of MMP1 was investigated after treatment of MDA-MB231 cells with apigenin and luteolin. 20 μM apigenin and 20 μM luteolin caused a significant and time-dependent down-regulation of MMP1 protein (**Figures 2A,B**).

**FIGURE 3:**
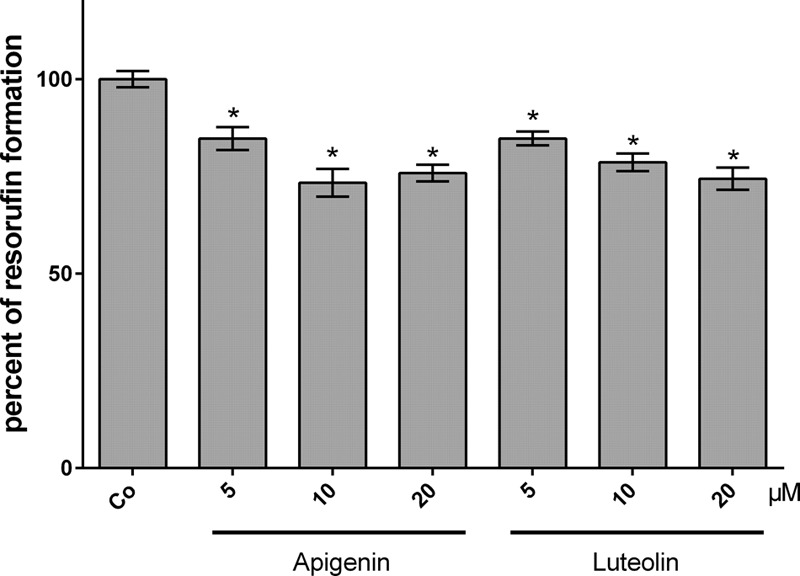
Inhibition of CYP1A1 activity in MDA-MB231 cells by flavonoids. MDA-MB231 cells were treated with solvent (DMSO, Co), or the indicated concentrations of apigenin and luteolin. Ethoxyresorufin (5 μM) was added after 4 h and the formation of resorufin was measured. Three independent experiments with at least three replicates were analyzed. Error bars indicate means ± SEM and asterisks significances (^∗^*p* < 0.05; ANOVA together with Tukey’s post-test).

Another mechanism of breast cancer emboli to cross the endothelial barrier is the secretion of 12(S)-HETE which, in MDA-MB231 cells, is produced by CYP1A1 (Nguyen et al., 2016a). Hence, the activity of CYP1A1 in MDA-MB231 cells was analyzed by EROD assay after treatment with increasing concentrations of apigenin and luteolin. 5–20 μM apigenin and luteolin inhibited CYP1A1 activity significantly within 4 h (**Figure 3**).

The results proved that apigenin and luteolin inhibited MMP1 expression and CYP1A1 activity, which trigger the retraction of the adjacent LEC barrier thereby enabling MDA-MB231 tumor spheroid intravasation.

### Influence of Apigenin and Luteolin on Intracellular Ca^2+^ Release in LEC

The intravasation of breast cancer cells relies on an interplay between cancer emboli and the LEC barrier (Vonach et al., 2011; Viola et al., 2013). Therefore, it was investigated whether apigenin or luteolin would also inhibit the pro-intravasative response of LECs.

**FIGURE 4:**
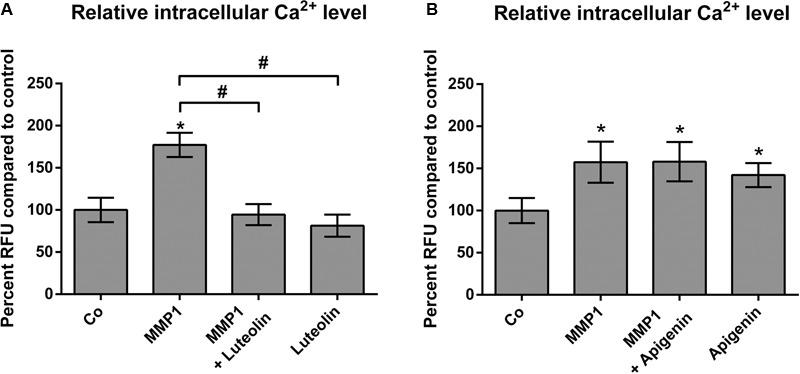
Modulation of MMP1-induced Ca^2+^ release by flavonoids in LECs. LECs (8 × 10^3^ cells/well) were pre-treated with solvent (DMSO, Co), or **(A)** 20 μM luteolin, or **(B)** 20 μM apigenin and then charged with FluoForte Dye-loading for 45 min at 37°C and 15 min at room temperature when cells were stimulated with 100 ng/mL activated recombinant MMP1 for 5 min. Intracellular free calcium was measured with a fluorescence plate reader at 490/525 nm. Three independent experiments with at least two replicates were analyzed. Error bars indicate means ± SEM, and asterisks and hash significances (*p* < 0.05; *t*-test).

**FIGURE 5:**
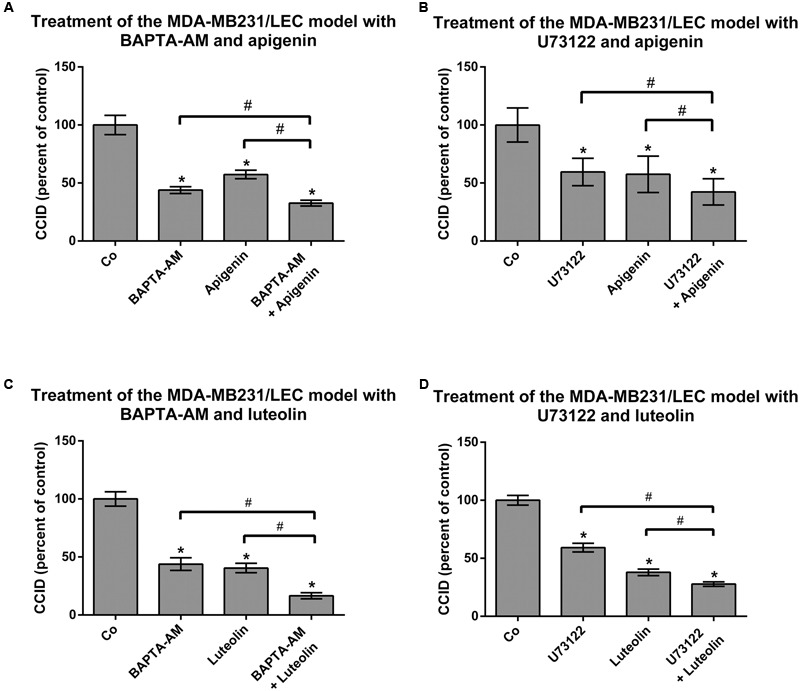
Analysis of drug combinations on CCID formation. MDA-MB231 spheroids were placed on LEC monolayers and treated with solvent (DMSO, Co), or **(A,B)** 20 μM apigenin, or **(C,D)** 20 μM luteolin, alone, or together with **(A,C)** 5 μM BAPTA-AM, or together with **(B,D)** 2.5 μM U73122 for 4 h, after which CCID areas were measured using an Axiovert microscope and Zen little 2012 software. Three independent experiments with at least five replicates were analyzed. Error bars indicate means ± SEM and asterisks and hash significances (^∗^*p* < 0.05; *t*-test).

The release of Ca^2+^ from intracellular stores plays a central role in MMP1- and 12(S)-HETE-induced CCID formation (Nguyen et al., 2016b; Stadler et al., 2017). Hence, it was tested whether apigenin and luteolin affected intracellular Ca^2+^ release. Luteolin prevented MMP1-induced Ca^2+^ increase (**Figure 4A**), which is consistent with previous reports (Jiang et al., 2005). In contrast, apigenin did not reduce MMP1-induced Ca^2+^ levels in LECs, but in contrast increased Ca^2+^ release (**Figure 4B**). This is in agreement with data that apigenin activates potassium channels leading to their hyper-polarization and causing the release of Ca^2+^ (Erdogan et al., 2007). Accordingly, treating the MDA-MB231/LEC model with apigenin and BAPTA-AM (intracellular Ca^2+^ inhibitor) caused a significantly stronger inhibition of CCID formation than apigenin alone, or BAPTA-AM alone (**Figure 5A**). Ca^2+^ release is dependent on PLCβ activity and, therefore, also the co-treatment with apigenin and U73122 (PLC inhibitor) attenuated CCID formation significantly stronger than the treatment with just apigenin or U73122 (**Figure 5B**). Luteolin ameliorated the CCID-inhibitory effect of BAPTA-AM (**Figure 5C**) or U73122 (**Figure 5D**) even more efficiently than apigenin. Overall, these data support the notion that an additional intravasative mechanism, which was independent of Ca^2+^ signaling, must have been inhibited by apigenin and luteolin as well.

### Luteolin/Apigenin Inhibit Phosphorylation of Tyr397-FAK in LEC

**FIGURE 6:**
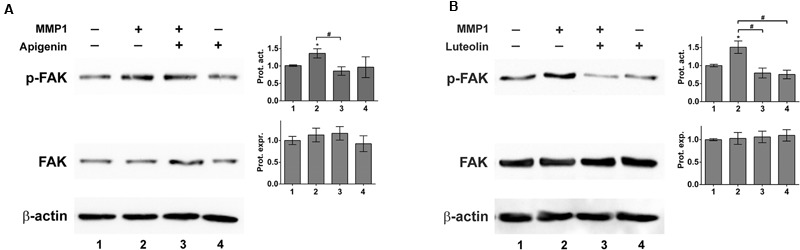
MMP1 activates focal adhesion kinase (FAK) in LECs. LECs were grown to ∼70–80% confluence and then pre-treated with solvent (DMSO), or **(A)** 20 μM apigenin, or **(B)** 20 μM luteolin and then stimulated with 100 ng/mL activated recombinant MMP1 for 4 h. Cells were lysed, proteins were separated by SDS gel electrophoresis and analyzed by Western blotting using the indicated antibodies. Equal sample loading was controlled by Ponceau S staining and β-actin immunoblotting. The relative protein expression (Prot. expr.) was quantified by densitometry facilitating the comparison with β-actin control, which was set to a value of “1.” The calculated FAK protein expression levels are shown in the bar graphs next to the FAK Western blots. The relative phosphorylation of FAK (p-FAK; corresponding to FAK activity) was quantified by densitometry facilitating the comparison with FAK protein expression, which was set to a value of “1.” The calculated FAK protein activity levels (Prot. Act.) are shown in the bar graphs next to the p-FAK Western blots. The numbers below the bar graphs refer to the respective treatment conditions indicated in the blot lanes. Densitometries are means ± SEM from at least three experiments (asterisks indicate significances, *p* < 0.05; *t*-test) and the Western blot images are representatives for illustration. Hashes (#; rhomboids) indicate significance.

**FIGURE 7:**
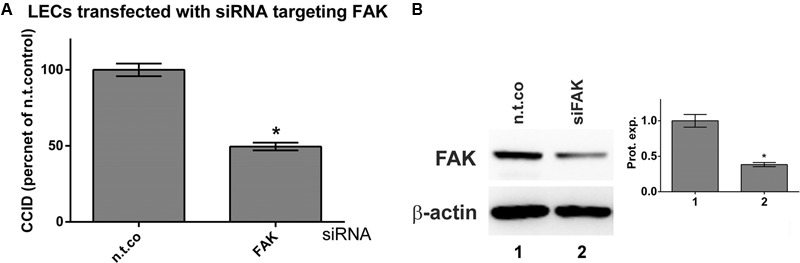
Inhibition of focal adhesion kinase (FAK) expression in LECs by specific siRNA. LECs were transiently transfected with either non-targeting siRNA (n.t.co) or siRNA inhibiting the expression of FAK. **(A)** MDA-MB231 spheroids were placed on confluent LEC monolayers and co-incubated for 4 h when the areas of CCIDs were analyzed using an Axiovert microscope and Zen Little 2012 software. Three independent experiments with at least five replicates were analyzed. **(B)** After 24 h cells were lysed, total protein was isolated, separated by SDS-PAGE and transferred to PVDF membranes for Western blotting using FAK antibody. β-Actin was used to control equal sample loading. Relative protein expression levels (Prot. expr.) are shown to the right of the blots and the numbers below the bar graphs refer to the respective treatment conditions shown below the lanes. Densitometries are means ± SEM from at least three experiments (asterisks indicate significances, *p* < 0.05; *t*-test) and the Western blot images are representatives for illustration.

The FAK controls cell-matrix interactions and mobility (Schwock et al., 2010) as observed in CCID formation (Nguyen et al., 2015). Recombinant MMP1 induced the phosphorylation of FAK at Tyr397 indicative for its activation. Pre-treatment with apigenin or luteolin prevented MMP1-induced, but not constitutive FAK phosphorylation (**Figures 6A,B**). The inhibition of Tyr397-FAK phosphorylation by apigenin did not correlate with the inhibition of Ca^2+^ release. Therefore, the relevance of FAK for CCID formation was studied by siRNA-mediated knock-down of FAK (siFAK). In fact, siFAK transfection of LECs suppressed MDA-MB231-induced CCID formation by ∼50% (**Figure 7A**). The knock-down of FAK was confirmed by Western blotting (**Figure 7B**). Hence, FAK significantly contributed to intravasation *in vitro* and apigenin and luteolin inhibited this mechanism.

**FIGURE 8:**
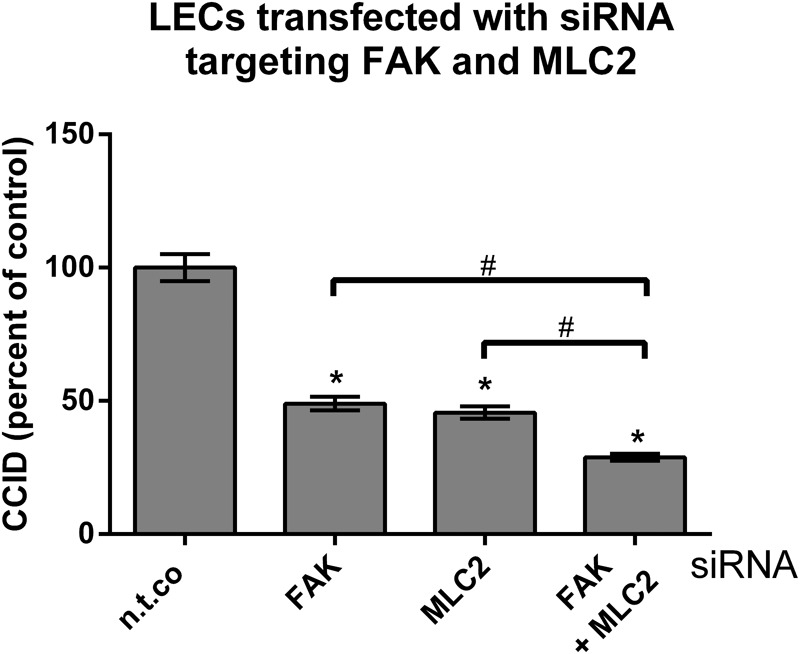
Inhibition of focal adhesion kinase (FAK) and myosin light chain 2 (MLC2) protein expression in LECs by specific siRNAs. LECs were transiently transfected with either non-targeting siRNA (n.t.co), or siRNAs inhibiting the expression of FAK and MLC2. Then, MDA-MB231 spheroids were placed on confluent LEC monolayers and co-incubated for 4 h, after which the areas of CCIDs were analyzed using an Axiovert microscope and Zen Little 2012 software. Three independent experiments with at least five replicates were analyzed. Error bars indicate means ± SEM, asterisks and hash significances (*p* < 0.05; *t*-test).

Earlier work demonstrated that CCID formation through MLC2 depends on intracellular Ca^2+^ release (Nguyen et al., 2015). Our results show that FAK-dependent CCID formation did not correlate with intracellular Ca^2+^ levels. Although both pathways were induced by MMP1, they, obviously, are independent of each other. Thus, we hypothesized that simultaneous inhibition of MLC2 (Ca^2+^-dependent) and FAK (Ca^2+^-independent) by respective siRNAs, should bring about a greater reduction in CCID formation than inhibition of either FAK or MLC2 alone, which was actually confirmed (**Figure 8**). This supports former data (**Figure 1C**) showing that luteolin (inhibiting FAK and Ca^2+^ release) and apigenin (inhibiting FAK but not Ca^2+^ release) synergize in CCID attenuation via distinct signaling pathways.

## Discussion

We show for the first time that luteolin and apigenin attenuate the intravasation of breast cancer cells *in vitro*. At the tested concentrations, luteolin and apigenin inhibited CYP1A1 activity in MDA-MB231 cells. CYP1A1 synthesizes the pro-metastatic arachidonic acid metabolite 12(S)-HETE and is induced, e.g., by the activated aryl hydrocarbon receptor, the expression of which is high in many cancer entities (Murray et al., 2014) and also in MDA-MB231 cells (Nguyen et al., 2016a). In HepG2 cells with a luciferase reporter fused to the CYP1A1-regulator region, 5 μM apigenin increased reporter gene expression by threefold. In contrast, the higher concentration of 20 μM apigenin inhibited the expression by 50% (Allen et al., 2001). In cell-free assays luteolin inhibited CYP1A1 more potently than apigenin, whereas CYP1A2 was inhibited more efficiently by apigenin (Kim et al., 2005). CYP1A2 mediates the formation of flavonoid metabolites with different properties to the genuin compounds. The comparability of flavonoid effects is rather difficult among different cell lines, which depend on the expression and activity profiles of the various CYP isoforms converting these flavonoids to other (more or less active) products (Androutsopoulos et al., 2009). Based on the very different individual expression levels of CYP1A2 in humans, Breinholt et al. (2002) conclude that this may result in different individual sensitivity.

Apigenin and luteolin inhibited also MMP1 expression in MDA-MB231 cells. MMP1 expression is induced by NF-κB (Lee et al., 2011), which is activated not only in inflamed- but also in cancerous tissue (Karin and Greten, 2005; Karin, 2006). Recently, we provided evidence that specific inhibition of NF-κB (by siRELA, siNFKB1, siNEMO) down-regulated MMP1 expression and attenuated CCID formation (Nguyen et al., 2015). Flavones such as apigenin and luteolin inhibited NF-κB activity and consequently the expression of its target MMP9 (Amrutha et al., 2014). Hence, the inhibition of NF-κB by apigenin and luteolin may have been the reason for suppressed MMP1 levels in MDA-MB231 cells.

As shown here, recombinant MMP1 significantly induced the phosphorylation of FAK at Tyr397 in LECs. FAK activity contributes to cancer cell migration by establishing loose cell-matrix interactions necessary for rapid cell movement (Desai et al., 2009; Schwock et al., 2010), as described in CCID formation (Kerjaschki et al., 2011). In our experiments suppression of FAK significantly inhibited CCID formation. The activation of FAK (through phosphorylation of Tyr397) is mediated through Kv2.1 channels (Wei et al., 2008). Since FAK as well as potassium channel activity contribute to LEC retraction and CCID formation (Nguyen et al., 2017), the reduction of Tyr397-FAK phosphorylation by apigenin may have been due to the inhibition of potassium channels. This was supported by reports demonstrating that trimethyl-apigenin interfered with cardiac potassium currents (Liu et al., 2012) and the inhibition of potassium channel activity by the flavonoids acacetin, scutellarin, genistein, and naringenin (Teisseyre and Michalak, 2005; Zhu et al., 2008; Wu et al., 2011; Gasiorowska et al., 2012). However, quite to the contrary, apigenin and luteolin exhibited vasodilatory effects, which are discussed to be due to activation (rather than inactivation) of potassium channels (Calderone et al., 2004; Jiang et al., 2005). A more recent research concluded that the vasodilatory properties of apigenin and luteolin were independent of potassium channel inhibition, but due to extracellular calcium influx (Roberts et al., 2013). Therefore, the mechanism by which apigenin and luteolin inhibit FAK phosphorylation remains to be established.

MMP1 also activates the mobility protein MLC2 in LECs, which contributes to CCID formation as well (Nguyen et al., 2015, 2016c). MMP1 binds to PAR1, activates PLCβ, triggers Ca^2+^ release and activates the Ca^2+^-calmodulin kinase MYLK, which phosphorylates MLC2. MMP1 was also shown to cause migration by binding to PAR1 in breast cancer cells (Boire et al., 2005) and blood endothelial cells (Juncker-Jensen et al., 2013). Whereas MMP1-induced MLC2 activation depends on Ca^2+^ release, FAK activation was not found to correlate with intracellular Ca^2+^ levels. This implicates that the pathway activating FAK represents a pro-intravasative mechanism independent of the one activating MLC2. Therefore, the inhibition both of FAK (either by siFAK or apigenin) and of MLC2 (by siMLC2, or BAPTA-AM, or U73122) reduced CCID formation in LEC monolayers to a greater extent than the inhibition of just one pathway.

Summing up, luteolin and apigenin inhibited pro-intravasative mechanisms in MDA-MB231 breast cancer cells at the levels of CYP1A1 activity and MMP1 expression. In LECs, apigenin and luteolin inhibited MMP1-induced, aberrant FAK activity but not constitutive, normal FAK phosphorylation. Only luteolin inhibited Ca^2+^ signaling in LEC, which contributed to CCID formation (Nguyen et al., 2016b). This may explain why luteolin affected CCID formation more potently than apigenin as well as the synergism between apigenin and luteolin.

The results implicate that luteolin and apigenin maintained the resilience of the endothelial barrier whilst attacking cancer cells and additionally, inhibited the malignant armamentarium of the tumor. Luteolin and apigenin exerted their effects at low μM concentrations which usually cannot be reached by daily dietary intake (Vogiatzoglou et al., 2015). Nevertheless, the daily intake of particular flavonoid subclasses (flavonols, flavan-3-ols, anthocyanidins) significantly correlates inversely with the risk of colorectal cancer (Woo and Kim, 2013). However, no other flavonoid subclasses or total flavonoids are associated with a lower risk of breast cancer in post-menopausal women (Hui et al., 2013). Intake of highly enriched apigenin and luteolin formulations achieved flavonoid concentrations in blood plasma which are comparable to those described herein *in vitro*. In detail, a single oral dose of apigenin delivered via a carbon nanopowder solid dispersion carrier (60 mg/kg) to rats resulted in peak plasma concentrations of up to 3.2 μg/mL which corresponds to 11.8 μM (Ding et al., 2014). Similarly, a single oral dose luteolin in a peanut hull extract (14.3 mg/kg) to rats resulted in peak plasma concentrations of up to 8.3 μg/mL (29.1 μM) (Zhou et al., 2008). The evaluation as to whether these concentrations are free of negative side effects throughout long term treatment requires controlled phase I studies.

## Author Contributions

JlH, AF, and DM performed the experiments. CN, NH, and SK analyzed the data. JqH, SG, PB, and WJ were involved in experiment planning and supervision. AÖ, LK, and GK supervised experimental work, and compiled and interpreted the data. LK and GK wrote the manuscript.

## Conflict of Interest Statement

The authors declare that the research was conducted in the absence of any commercial or financial relationships that could be construed as a potential conflict of interest.
